# ATP synthase subunit alpha and LV mass in ischaemic human hearts

**DOI:** 10.1111/jcmm.12477

**Published:** 2014-11-09

**Authors:** Esther Roselló-Lletí, Estefanía Tarazón, María G Barderas, Ana Ortega, Maria Micaela Molina-Navarro, Alba Martínez, Francisca Lago, Luis Martínez-Dolz, Jose Ramón González-Juanatey, Antonio Salvador, Manuel Portolés, Miguel Rivera

**Affiliations:** a Cardiocirculatory Unit, Health Research Institute Hospital La FeValencia, Spain; b Department of Vascular Physiopathology, National Hospital of Paraplegics, SESCAMToledo, Spain; c Cellular and Molecular Cardiology Research Unit, Department of Cardiology and Institute of Biomedical Research, University Clinical HospitalSantiago de Compostela, Spain; d Cardiology Service, Hospital La FeValencia, Spain; e Cell Biology and Pathology Unit, Health Research Institute Hospital La FeValencia, Spain

**Keywords:** heart failure, ischaemic cardiomyopathy, mitochondria, tissue, transplantation

## Abstract

Mitochondrial dysfunction plays a critical role in the development of ischaemic cardiomyopathy (ICM). In this study, the mitochondrial proteome in the cardiac tissue of ICM patients was analysed by quantitative differential electrophoresis (2D-DIGE) and mass spectrometry (MS) for the first time to provide new insights into cardiac dysfunction in this cardiomyopathy. We isolated mitochondria from LV samples of explanted hearts of ICM patients (*n* = 8) and control donors (*n* = 8) and used a proteomic approach to investigate the variations in mitochondrial protein expression. We found that most of the altered proteins were involved in cardiac energy metabolism (82%). We focused on ATPA, which is involved in energy production, and dihydrolipoyl dehydrogenase, implicated in substrate utilization, and observed that these molecules were overexpressed and that the changes detected in the processes mediated by these proteins were closely related. Notably, we found that ATPA overexpression was associated with reduction in LV mass (*r* = −0.74, *P* < 0.01). We also found a substantial increase in the expression of elongation factor Tu, a molecule implicated in protein synthesis, and PRDX3, involved in the stress response. All of these changes were validated using classical techniques and by using novel and precise selected reaction monitoring analysis and an RNA sequencing approach, with the total heart samples being increased to 24. This study provides key insights that enhance our understanding of the cellular mechanisms related to the pathophysiology of ICM and could lead to the development of aetiology-specific heart failure therapies. ATPA could serve as a molecular target suitable for new therapeutic interventions.

## Introduction

Heart failure (HF) is a leading cause of morbidity and mortality in industrialized countries, and ischaemic cardiomyopathy (ICM) is one of the main causes of this syndrome 1. Advances in the understanding of the molecular basis of myocardial ischaemia have yielded deep insights into the complex sequence of events occurring in ICM, regardless of its manifestations. This enhanced understanding has highlighted the critical role played by cardiac mitochondria in this cardiomyopathy 2,3.

Several key studies have focused on how mitochondria contribute to the development and progression of HF, because mitochondria play a central role in energy production, metabolism, calcium homoeostasis and oxidative stress 4–9. Alterations in mitochondrial bioenergetics appear to be critically involved in ICM. The failing heart exhibits marked mitochondrial abnormalities that impair the ability of the cardiac tissue to synthesize ATP. Thus, impaired oxidative phosphorylation can reduce cardiac function by providing an insufficient supply of ATP to cardiac myocytes 7. Although promising mitochondrion-targeted drugs have emerged, successful clinical trials have been completed for only very few of these drugs. Therefore, the mitochondrion remains a potential untapped target for use in new ICM therapies. Mitochondria have been studied extensively using experimental models and conventional biochemical methods 10–12. These studies have typically focused on only one particular protein rather than on the entire cardiac mitochondrial proteome. Furthermore, the mitochondrial proteome has not been analysed in pathological human hearts by 2D-DIGE and MS/MS. Thus, characterizing the mitochondrial proteome could provide new insights into cardiac dysfunction and suggest new molecular targets for use in therapeutic interventions designed for ICM.

In this study, we isolated mitochondria from LV samples of explanted human hearts of patients with ICM and used a proteomic approach to investigate the variations in mitochondrial protein expression. Our results reveal that, in ischaemic human hearts, proteins involved mainly in energy metabolism and also those involved in protein synthesis and stress response increased. We focused on four representative mitochondrial proteins that were altered in these processes, which were validated using distinct classical techniques as well as novel and precise selected reaction monitoring (SRM) analysis and an RNA sequencing (RNAseq) approach. We found that these proteins could play a key role in ICM. Specifically, our results indicate a direct relationship between the overexpression of ATP synthase subunit alpha (ATPA) and a reduction in LV mass (LVM).

## Materials and methods

### Tissue sources

Experiments were performed with LV samples obtained from the explanted hearts of Caucasian patients who had ICM and had undergone cardiac transplantation (*n* = 16). Clinical history, haemodynamic study, electrocardiography and Doppler echocardiography data were available for all the patients. All of the patients were functionally classified based on according to the New York Heart Association (NYHA) criteria, and the patients received medical treatment following the guidelines of the European Society of Cardiology 13.

All controls (*n* = 8) had normal LV function (EF >50%), as determined by Doppler echocardiography, and no history of cardiac disease. The control (CNT) samples were obtained from non-diseased donor hearts that had been rejected for cardiac transplantation owing to size or blood type incompatibility, and because of the impossibility of finding a new recipient during the set period for transplant programme. For these donors, the cause of death was either cerebrovascular or motor vehicle accidents.

To collect that amount of control hearts that somehow means that its therapeutic usefulness as original donors objective, has been changed to research use in the last moment because unexpected circumstances previously described (never because explanted malfunction), we have been collecting heart samples right in the Operating Room for 12 years, attending more than the 90% of all heart transplant procedures, to care for heart samples appropriateness.

Tissue samples were obtained, by our choice because we have access to the full explanted heart, from near the apex of the left ventricle, were maintained in 0.9% NaCl and were preserved at 4°C for an exceptional maximum of 6 hrs after the loss of coronary circulation. The samples were stored at −80°C until they were used to isolate mitochondria. Of the 24 heart samples, 16 were used for proteomic analysis (ICM, *n* = 8; CNT, *n* = 8). All 24 heart samples were used in the validation study to improve the numerical base by increasing the number of patients (ICM, *n* = 16; CNT, *n* = 8).

This study was approved by our Institution Ethics Committee (Biomedical Investigation Ethics Committee). Signed informed consent was obtained from each patient prior to tissue collection. The investigation was conducted in accordance with the guidelines of the Declaration of Helsinki 14.

### Mitochondrial isolation and proteomic analysis

Mitochondria were isolated using standard homogenization, protease digestion and differential centrifugation methods, as previously described by Imahashi *et al*. 15. Two-dimensional electrophoresis, 2D-DIGE, in-gel digestion of proteins, sample preparation for mass spectrometry (MS) and MALDI-MS (/MS), and database searching have been described in Supporting information.

### Gel electrophoresis and Western blot analysis

Protein samples used for detecting ATPA, dihydrolipoyl dehydrogenase (DLDH), elongation factor Tu (EFTU) and thioredoxin-dependent peroxide reductase (PRDX3) were separated using Bis-Tris electrophoresis on 4–12% polyacrylamide gels under reducing conditions. After electrophoresis, proteins were transferred from the gels to polyvinylidene difluoride membranes by using an iBlot Dry Blotting System (Invitrogen Ltd., Manchester, UK) for use in Western blot analyses. The primary antibodies used for detection were anti-ATP5A mouse monoclonal antibody (1:300), anti-lipoamide dehydrogenase rabbit monoclonal antibody (1:1000), anti-TUFM rabbit polyclonal antibody (1:2000) and anti-peroxiredoxin 3 mouse monoclonal antibody (1:1000; all obtained from Abcam, Cambridge, UK). To control for protein loading 16, we used an anti-COX IV rabbit polyclonal antibody (1:200; Thermo Scientific, Rockford, IL, USA).

Immunoreactive protein bands were visualized using an acid phosphatase-conjugated secondary antibody and nitro blue tetrazolium/5-bromo-4-chloro-3-indolyl phosphate (NBT/BCIP, Sigma-Aldrich, St. Louis, USA) substrate system. The bands were digitized using an image analyser (DNR Bio-Imagining Systems, Jerusalem, Israel) and quantified using the GelQuant Pro (v12.2) program.

### Fluorescence microscopy

Human myocardial LV samples were fixed in 4% formalin, embedded in paraffin, cut into 5-μm sections, and mounted on SuperFrost glass slides. Sections were maintained at 60°C overnight, deparaffinized using xylol, and then washed in 100%, 96%, 80% and 70% ethanol. Next, the samples were blocked with PBS containing 1% bovine serum albumin (BSA) for 15 min. at room temperature (RT). After blocking, the sections were incubated for 120 min. at RT with primary antibodies (described in the ‘Western blot analysis’ section above) diluted in the blocking buffer and were then incubated with Alexa-conjugated secondary antibodies (Invitrogen, New York, USA) for 60 min. at RT 17. Finally, the sections were rinsed in PBS, mounted in Vectashield-conjugated 4′,6-diamidino-2-phenylindole (DAPI) to identify nuclei (Vector Laboratories, Burlingame, CA, USA), and examined under an Olympus BX50 fluorescence microscope (Tokyo, Japan). The images were processed using the ImageJ software (v. 1.46r; National Institutes of Health, Bethesda, MD, USA).

### Immunocytochemistry and electron microscopy

Myocardial samples (size 1 mm^3^) prepared from the LV tissues were fixed in a solution of 1.5% glutaraldehyde and 1% formaldehyde in 0.05 M cacodylate buffer (pH 7.4) for 1 hr at 4°C. The samples were then post-fixed in 1% OsO_4_ for 1 hr at 4°C, dehydrated in ethanol, and embedded in Epon 812. Ultrathin sections (80 nm) were obtained and mounted on nickel grids and counterstained with 2% uranyl acetate for 20 min. and 2.7% lead citrate for 3 min. 18,19.

To perform immunogold labelling, ultrathin sections were floated for 30 min. on 0.1% BSA-Tris buffer (20 mM Tris-HCl, 0.9% NaCl, pH 7.4, containing 0.1% BSA, Type V) and for 2 hrs in a moist chamber at RT on sodium metaperiodate 20. After rinsing with bi-distilled water, the sections were incubated for 5 min. with 3% H_2_O_2_. The grids were rinsed again with bi-distilled water and incubated separately in a moist chamber overnight at RT with primary antibodies (described in the ‘Western blot analysis’ section above) diluted in the 0.1% BSA-Tris buffer. After rinsing with 0.1% BSA-Tris buffer, the sections were incubated in a moist chamber for 1 hr at 37°C with 0.1% BSA-Tris buffer containing 0.05% Tween-20 and either goat anti-rabbit IgG-gold antibodies (10 nm, 1:10 dilution; Sigma-Aldrich) to detect DLDH and EFTU or goat antimouse IgG-gold antibodies (5 nm, 1:10 dilution; Sigma-Aldrich) to detect ATPA and PRDX3.

After the sections were rinsed with 0.1% BSA-Tris buffer and bi-distilled water, they were air-dried and counterstained first with uranyl acetate for 30 min. and then with lead citrate for 5 sec. Finally, the grids were air-dried completely. To perform electron microscopy, a Philips CM-100 system (Amsterdam, Netherlands) was used, with magnifications ranging from 4500× from 15,000×.

### Selected reaction monitoring and RNA sequencing analysis

Methods used for SRM, RNA extraction, RNAseq and computational analysis of the RNAseq data are included as Data S1.

### Statistics

Data are presented as mean ± SD. The Kolmogorov–Smirnov test was used to analyse the normal distribution of the variables. Comparisons between two groups were performed with Student's *t*-test, whereas Pearson's correlation coefficient was calculated to analyse the association between variables. Analyses were considered significant at *P* < 0.05. All statistical analyses were performed with the SPSS Software v. 20 for Windows (IBM SPSS Inc., Chicago, IL, USA).

## Results

### Patients’ clinical characteristics

LV tissue samples were obtained from 16 patients with ICM (81% men; mean age, 55 ± 8 years; ejection fraction, <40%). These patients had an NYHA functional classification of III–IV and had been previously diagnosed with significant comorbidities, including hypertension, hypercholesterolaemia, obesity and diabetes mellitus. The patients’ clinical and echocardiographic characteristics are summarized in Table1. Eight non-diseased donor hearts were used as CNT samples (63% men; mean age, 55 ± 8 years; ejection fraction, >50%).

**Table 1:** Clinical and echocardiographic characteristics of patients with ischaemic cardiomyopathy

	ICM (*n* = 16)
Age (years)	55 ± 8
Gender male (%)	81
BMI (kg/m^2^)	27 ± 4
Prior hypertension (%)	50
Diabetes mellitus (%)	46
NYHA class	3.2 ± 1
Haemoglobin (mg/dl)	12 ± 3
Haematocrit (%)	37 ± 8
Total cholesterol (mg/dl)	143 ± 49
Duration of disease (months)	51 ± 47
Echo-Doppler study	
Ejection fraction (%)	25 ± 6
Fractional shortening (%)	14 ± 2
LV end-systolic diameter (mm)	53 ± 6
LV end-diastolic diameter (mm)	62 ± 8
Left ventricle mass (g)	277 ± 64
Left ventricle mass index (g/cm^2^)	134 ± 37

Duration of disease from diagnosis of heart failure until heart transplant. BMI, body mass index; ICM, ischaemic cardiomyopathy; NYHA, New York Heart Association.

### Differentially expressed mitochondrial proteins in patients with HF of ischaemic aetiology

The protein expression profiles of purified cardiac mitochondria obtained from eight ICM patients and eight CNT donors were compared using two-dimensional differential gel electrophoresis (2D-DIGE). Each gel contained the mitochondrial proteome samples of ICM patients, CNT samples and an internal standard. Gel images were imported into the DeCyder Differential Analysis Software, which detected 1418 protein spots. Reproducibility was tested by comparing the variation between distinct gels in the same group; no significant differences were detected using the *t*-test statistical analysis. We focused on identifying the up- and down-regulation of spot intensities where the fold-change was ≥1.5 (*P* < 0.05). Considering these criteria, the statistical analysis of the data performed with the DeCyder software revealed changes in the abundance of 12 protein spots that corresponded to 11 mitochondrial proteins (Fig. S1), which were identified using MS. We found that 10 spots were significantly up-regulated and two were down-regulated in ICM hearts. The details of tandem MS (MS/MS) identification of proteins are summarized in Table S1.

Table2 shows that in the mitochondrial proteome of ICM patients, the proteins that were altered are localized mainly in the mitochondrial matrix or the inner membrane. Most of these proteins are involved in cardiac energy metabolism (82%), with some being implicated in energy production, such as ATPA, and others being implicated in substrate utilization, such as DLDH. The remaining mitochondrial proteins identified here were a structural protein, coiled-coil-helix-coiled-coil-helix domain-containing protein 3; a protein implicated in protein synthesis, EFTU; and a protein involved in stress response, PRDX3.

**Table 2:** Mitochondrial proteins differentially regulated in ischaemic cardiomyopathy *versus* controls

Spot	Accession code	Protein	Fold-change	*P*-value	Main localization	Function
219	ECHA_HUMAN	Trifunctional enzyme subunit alpha	−1.69	0.040	Mitocondrion	Metabolism
344	ETFD_HUMAN	Electron transfer flavoprotein-ubiquinone oxidoreductase	+1.51	0.046	Matrix/Inner membrane	Metabolism/Transport
348	DLDH_HUMAN	Dihydrolipoyl dehydrogenase	+2.09	0.030	Matrix	Metabolism
361	AL4A1_HUMAN	Delta-1-pyrroline-5-carboxylate dehydrogenase	+1.58	0.039	Matrix	Metabolism
407	DLDH_HUMAN	Dihydrolipoyl dehydrogenase	+1.63	0.030	Matrix	Metabolism
464	ATPA_HUMAN	ATP synthase subunit alpha	+1.69	0.026	Inner membrane	Metabolism/respiratory chain
472	ODO2_HUMAN	Dihydrolipoyllysine-residue succinyltransferase component of 2-oxoglutarate dehydrogenase complex	+1.62	0.043	Matrix	Metabolism
614	KCRS_HUMAN	Creatine kinase S-type	−1.24	0.010	Inner membrane	Metabolism
689	EFTU_HUMAN	Elongation factor Tu	+1.56	0.049	Mitochondrion	Protein biosynthesis
882	ATPA_HUMAN	ATP synthase subunit alpha	+1.89	0.006	Inner membrane	Metabolism/Respiratory chain
960	CHCH3_HUMAN	Coiled-coil-helix-coiled-coil-helix domain-containing protein 3	+1.74	0.011	Inner membrane	Structural
1019	PRDX3_HUMAN	Thioredoxin-dependent peroxide reductase	+1.88	0.017	Mitochondrion	Stress response
	NDUV2_HUMAN	NADH dehydrogenase [ubiquinone] flavoprotein 2			Inner membrane	Metabolism/Respiratory chain

### Validation of differential protein abundance and mRNA levels of ATPA and DLDH

**Fig 1:**
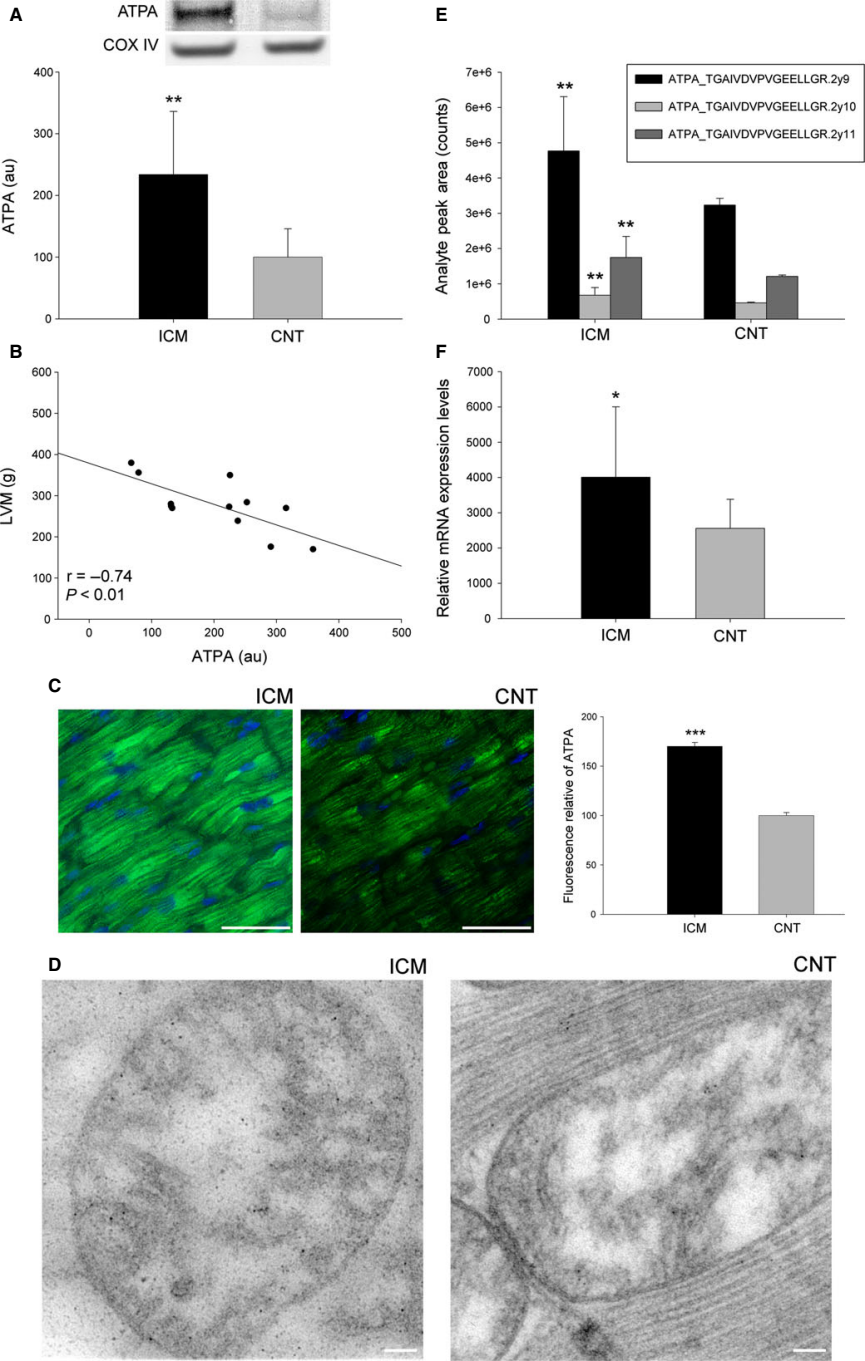
Validation of ATPA overexpression (energy production) and its relationship with LVM in ICM patients. (A) The influence of ICM on the amount of ATPA analysed using Western blotting techniques. The values of the controls were set as 100. Values were normalized relative to COX IV and finally to the CNT group. The data are expressed as mean ± SD in arbitrary units (optical density). (B) Scatter plots showing the relationship between ATPA protein levels and LV mass. (C) ATPA protein overexpression in ischaemic human hearts analysed using immunofluorescence techniques. The nuclei are shown co-stained with DAPI (blue), and the bar represents 100 μm. The bar graph shows the relative fluorescence intensity in ischaemic compared to control hearts. (D) ATPA localization and overexpression in ischaemic human hearts analysed using transmission electron microscopy. The bar represents 100 nm. (E) SRM validation of ATPA by using LC-MS/MS. (F) Levels of mRNA expression determined using RNAseq. Images are representative of the results obtained for all ICM patients and CNT donors included in the study. All data are expressed as mean ± SD. ATPA, ATP synthase subunit α; CNT, control; ICM, ischaemic cardiomyopathy; LVM, LV mass. *P < 0.05, **P < 0.01, ***P < 0.0001.

**Fig 2:**
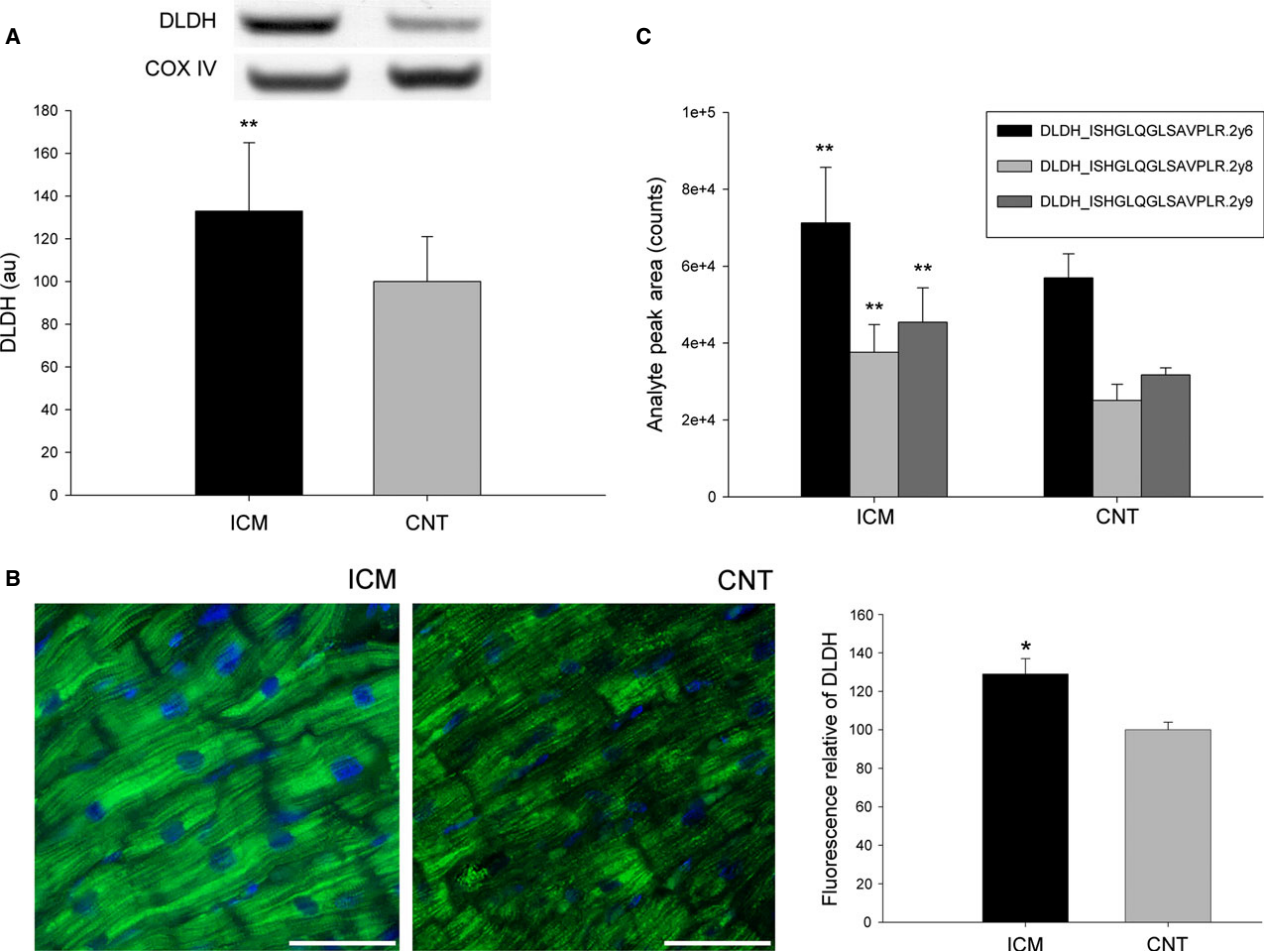
Validation of DLDH overexpression (substrate utilization). (A) The influence of ICM on the amount of DLDH analysed using Western blotting techniques. The values of the controls were set as 100. Values were normalized relative to COX IV and finally to the CNT group. The data are expressed as mean + SD in arbitrary units (optical density). (B) DLDH protein overexpression in ischaemic human hearts analysed using immunofluorescence techniques. The nuclei are shown co-stained with DAPI (blue), and the bar represents 100 μm. The bar graph shows the relative fluorescence intensity in ischaemic compared to control hearts. (C) SRM validation of DLDH by using LC-MS/MS. Images are representative of the results obtained for all ICM patients and CNT donors included in the study. All data are expressed as mean ± SD. CNT, control; DLDH, dihydrolipoyl dehydrogenase; ICM, ischaemic cardiomyopathy. *P < 0.05, **P < 0.01.

We focused on ATPA and DLDH as representative proteins of the main components of the metabolic machinery that function in energy production and substrate utilization, respectively, in addition to its described implication in HF 21. The changes in these proteins were validated using distinct techniques that compared the levels of ATPA and DLDH among ICM patients (*n* = 16) and CNT donors (*n* = 8). First, using Western blotting, we found the levels of these two proteins involved in metabolism. As shown in Figures1A and 2A, the levels of the analysed molecules were significantly increased in the pathological samples (ATPA, 234 ± 102 au *versus* 100 ± 46 au, *P* < 0.01; DLDH, 133 ± 32 au *versus* 100 ± 21 au, *P* < 0.05). These results coincided with those of the proteomic analysis.

Next, we found whether the levels of these proteins were related to specific image-derived parameters presented in Table1. The LV function parameters were completely available in 12 of 16 samples obtained from ICM patients. We found a highly significant inverse relationship between ATPA and LV mass (*r* = −0.73, *P* < 0.01) (Fig.1B). A multivariate linear regression analysis was used to test the independent predictive power of ATPA (adjusted for age and gender) on LVM in ischaemic patients. ATPA is an independent factor associated with LVM (*P* < 0.05, *r*^2^ = 0.68). However, DLDH did not reach statistical significance when we correlated it with this parameter.

The findings of our immunofluorescence studies agreed with the increased levels of the validated proteins detected using Western blotting and proteomic analysis: the fluorescence intensity of the validated proteins was higher in the ischaemic hearts than in the CNT samples. As shown in Figures1C and 2B, these proteins were diffusely distributed in the cytoplasm, with the fluorescence signal being substantially higher in the ischaemic group than in the CNT group. Immunocytochemistry studies confirmed the results of our aforementioned analyses and also the localization and distribution of these mitochondrial proteins. As shown in Figure1D, immunogold labelling of ATPA in ICM hearts was elevated compared with that in CNT hearts. We also confirmed the localization of ATPA and observed that this protein was similarly distributed in pathological and control samples. Similar results were obtained for DLDH (data not shown).

To validate the previous analyses and to evaluate the possible relationship between these altered proteins involved in metabolic processes, ATPA and DLDH were monitored using SRM (Figs1E and 2C, Table S2). Differential expression was confirmed in all three transitions per peptide (ATPA, *P* < 0.01; DLDH, *P* < 0.01), and, furthermore, the abundances of these altered proteins involved in energy production and substrate utilization were strongly correlated (ATPA *versus* DLDH, *r* = 0.70, *P* < 0.01).

Next, we used RNAseq to determine the mRNA differences between ICM patients and CNT donors. The mRNA levels of the ATPA gene (*ATP5A1*) were higher in the ICM group than in the control (57-fold, *P* < 0.05) (Fig.1F). These results also agree with those of the proteomic analysis, demonstrating the same trend in gene expression and protein levels. However, the expression of the DLDH gene (*DLD*) did not reach statistical significance.

### Validation of differential protein abundance and mRNA levels of EFTU and PRDX3

**Fig 3:**
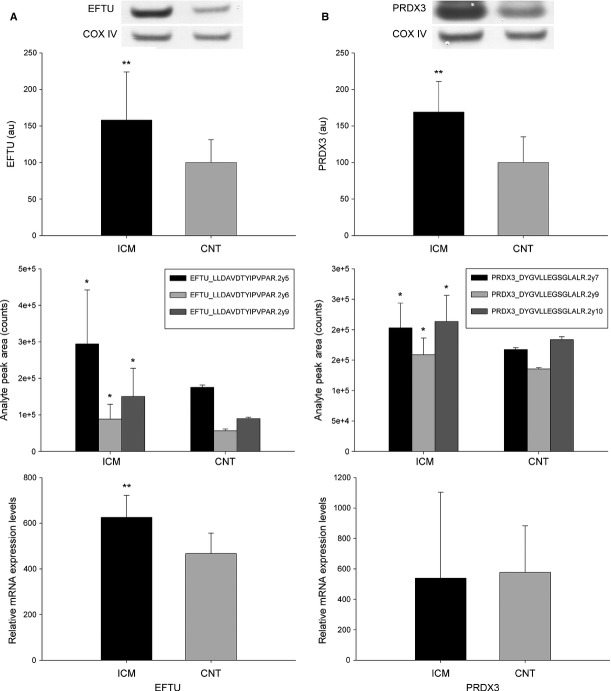
Validation of the overexpression of EFTU (protein biosynthesis) and PRDX3 (stress response). (A) The influence of ICM on the amounts of EFTU analysed using Western blotting techniques. The values of the controls were set as 100. Values were normalized relative to COX IV and finally to the CNT group. The data are expressed as mean ± SD in arbitrary units (optical density). SRM validation of EFTU by using LC-MS/MS. Levels of mRNA expression of EFTU determined using RNAseq. (B) The influence of ICM on the amounts of PRDX3 analysed using Western blotting techniques. The values of the controls were set as 100. Values were normalized relative to COX IV and finally to the CNT group. The data are expressed as mean ± SD in arbitrary units (optical density). SRM validation of PRDX3 by using LC-MS/MS. Levels of mRNA expression of PRDX3 determined using RNAseq. All data are expressed as mean ± SD. CNT, control; EFTU, elongation factor Tu; PRDX3, thioredoxin-dependent peroxide reductase, ICM, ischaemic cardiomyopathy. *P < 0.05, **P < 0.01.

We also validated the alteration of a protein implicated in protein synthesis, EFTU (Fig.3A), and of a protein involved in stress response, PRDX3 (Fig.3B). Western blot and SRM analyses (Table S2) showed a marked increase in the expression of EFTU and PRDX3 in ICM hearts, and immunofluorescence and immunocytochemistry studies revealed the same tendency in both proteins (data not shown). These results coincided with those of the proteomic analysis. Moreover, we determined the mRNA differences between ICM patients and CNT donors using RNAseq: the mRNA levels of the EFTU gene (*TUFM*) were higher than those for the control in the ICM group (34-fold, *P* < 0.01). However, we did not find significant differences in the case of the PRDX3 gene (*PRDX3*).

## Discussion

In this study, we performed 2D-DIGE analyses on cardiac mitochondria isolated from the LV tissue of ICM patients to investigate the changes in cardiac mitochondrial protein expression. Mitochondrial dysfunction plays a key role in the development of this cardiomyopathy 2,3. However, the mechanisms responsible for mitochondrial alterations in human hearts are poorly understood, and the animal models used to elucidate these mechanisms may not reflect the true pathophysiology of ICM. To our knowledge, this is the first study to analyse the mitochondrial proteome in pathological human hearts. Focusing research efforts on targeting mitochondrial dysfunction in the failing heart is crucial for restoring the myocardium and its contractile function. Thus, analysing the mitochondrial proteome could provide new insights into cardiac dysfunction in ICM patients. Here, we identified 12 protein spots corresponding to 11 mitochondrial proteins that were altered in failing hearts; nine proteins were increased, two were decreased. These changes comprise numerous aspects of mitochondrial function, but most of the altered proteins (9 of the 11 differentially regulated proteins, 82%) are involved in energy metabolism, which suggests that these are the proteins that are most sensitive to myocardial ischaemic injury.

Mitochondrial oxidative phosphorylation forms the basis of ATP production 7. Cardiac work is supported by a high rate of ATP hydrolysis, which is matched by ATP production through mitochondrial oxidative phosphorylation. During HF development, both energy demands and metabolism change cause a drastic reduction in oxidative phosphorylation and a shift towards glucose over fatty-acid utilization 22. Moreover, high rates of myocardial energy production are required to maintain the constant ATP demand of the working heart, and alterations in oxidative phosphorylation reduce cardiac function by providing an insufficient supply of ATP to cardiomyocytes 7. Our data show specific alterations in the ATP synthase system in ICM patients, we observed a substantial overexpression of ATPA. An interesting functional analysis in rats published by Wang *et al*. showed that inhibition of mitochondrial ATP synthase abolished the intermittent improvements induced by hypobaric hypoxia, specifically reducing post-ischaemic recovery of LV function, mitochondrial membrane potential and respiratory control ratios 23. A critical finding of our study was the close relationship between ATPA protein level and LV mass, which indicates that an increase in the protein level is associated with a reduction in LV mass. Thus, our results have once again demonstrated that ATP synthase activity is a key player in cardioprotection and have revealed for first time a direct relationship between the level of ATPA and the degree of hypertrophy in human ischaemic cardiac tissue. Taken together, these results imply that the mitochondrial ATP synthase could potentially be targeted in therapies to protect the heart against ischaemic injury and remodelling. To verify the causality of this highly significant relationship, additional studies are required.

We also found a strong positive correlation between the protein levels of ATPA and DLDH, a protein that is involved in substrate utilization. This correlation highlights the relationship between the two principal components of the cardiac energy metabolism system, energy production and substrate utilization. We found overexpression of DLDH, a stable homodimer that is an essential component of the pyruvate-dehydrogenase and glycine-cleavage systems, as well as of the α-ketoacid dehydrogenase complex 24. This result agrees with those published by our group and also by Li *et al*. in previous studies conducted using total homogenates of LV tissue of ICM patients, which revealed increased expression of DLDH 21,25.

Oxidative stress has been widely reported to be a crucial feature in the pathophysiology and development of HF occurring through free-radical production 26,27. Reactive oxygen species play a key role in the onset and progression of coronary heart disease, tissue necrosis, and contractile dysfunction 28,29. PRDX3 is a mitochondrial antioxidant protein that protects radical-sensitive enzymes against oxidative damage by means of a radical-generating system. PRDX3 overexpression has been reported to protect the heart against post-myocardial infarct remodelling and failure in mice by reducing LV cavity dilation, dysfunction, fibrosis and apoptosis 30. These results are consistent with our findings, because we found that PRDX3 levels were increased substantially in the cardiac tissue of ICM patients. Furthermore, we found that PRDX3 levels were strongly correlated with those of other altered proteins that are implicated in protein synthesis, specifically EFTU, a result that interconnects the changes occurring in distinct processes. Thus, oxidative damage is linked to increased activation of protein biosynthesis in failing hearts, because EFTU promotes the GTP-dependent binding of aminoacyl-tRNA to the A-site of ribosomes, and the down-regulation of EFTU increases reactive oxygen species 31.

A common limitation of studies in which the cardiac tissues used are obtained from end-stage failing human hearts is that disease aetiology and treatments vary considerably. However, we ensured that our study population was aetiologically homogeneous. Moreover, our tissue samples were obtained from the transmural LV apex, and thus our findings cannot be generalized to all layers and regions of the LV. However, a crucial factor of our study was that it was conducted using a large number of samples collected from explanted hearts of patients who had undergone cardiac transplantation, which makes our results applicable to the ICM population.

In summary, this study is the first to analyse the mitochondrial proteome in the cardiac tissue of ICM patients. We found significant and reproducible alterations in cardiac energy metabolism, especially in molecules involved in substrate utilization and energy production, which revealed a close relationship between the changes detected in these two processes. Importantly, we observed that ATPA overexpression was strongly correlated with a reduction in LV mass. This work provides new insights into the cellular mechanisms associated with the pathophysiology of ICM and could serve as a pivotal study in the development of aetiology-specific HF therapies. Our data identify ATPA as a molecule that could be targeted in new therapeutic interventions developed for ICM patients.

## Supporting Information

**Data S1** Two-dimensional electrophoresis, 2D-DIGE, in-gel digestion of proteins, sample preparation for mass spectrometry (MS) and MALDI-MS (/MS), and database searching have been described in as Data S1. In addition, methods used for SRM, RNA extraction, RNAseq and computational analysis of the RNAseq data are included as Data S1. The details of tandem MS (MS/MS) identification of proteins and SRM are summarized in Table S1 and S2. Figure S1 shows a representative two-dimensional DIGE gel.
