# Supplementary material for: ATP synthase subunit alpha and LV mass in ischaemic human hearts
Source: J Cell Mol Med. 2014 Nov 9;19(2):442–51. doi: 10.1111/jcmm.12477 (PMC4407605; doi:10.1111/jcmm.12477)
Supplement: Supplementary file 1 [file jcmm0019-0442-sd1.doc]

**SUPPORTING INFORMATION**

**Supplemental Figure 1. Representative two-dimensional DIGE gel.** A differential abundance analysis of the ischemic hearts proteome was performed by saturation labeling two-dimensional-DIGE analysis. Assayed comparisons were: pathological samples *versus* control (ICM *versus* CNT). Spots found significantly varied (*p value* < 0.05) and with a fold change greater than 1.5 or lower than -1.5, are represented in a representative two-dimensional DIGE gel.

**
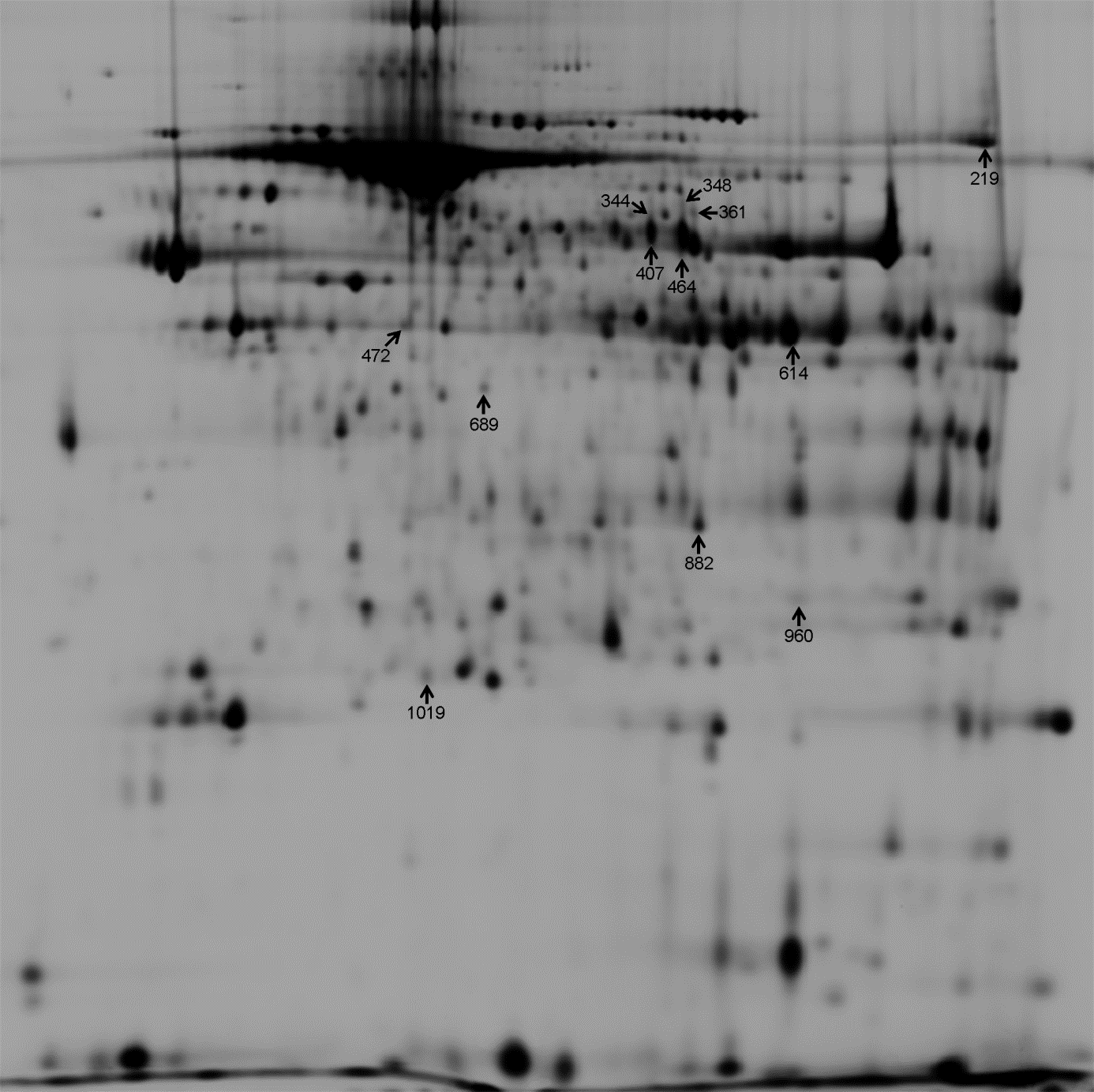
**

**SUPPLEMETAL METHODS**

**2D-DIGE**

Protein samples were precipitated using a 2-D Clean-Up Kit (GE Healthcare) as per the manufacturer’s protocol. Samples were labeled using CyDye DIGE Fluor Minimal Dyes (GE Healthcare) according to the manufacturer’s recommendations. The samples were resuspended in 20 μL of labeling buffer containing 7 M urea, 2 M thiourea, 4% CHAPS, and 30 mM Tris. The pH was then checked in every sample to ensure an optimal labeling reaction (pH 8.0–9.0). Aliquots (50 μg protein in 10 μL) were separated into individual tubes and a pooled internal standard was generated by mixing of equal amounts of all samples included in the experiment. A total of 400 pmol of the appropriate CyDye was added to each sample according to the experimental protocol. This experimental design included 2 experimental groups and 8 samples per group, and thus 16 samples were labeled with either Cy3 or Cy5. The labeling reactions were developed for 30 min on ice in the dark and then quenched by incubation with 1 μL of 10 mM lysine for 10 min in the dark. The labeled samples were combined according to the experimental design and loaded onto the same immobilized pH-gradient (IPG) strip. We randomized the CyDye assignments and sample combinations in each gel, which also included one aliquot of the pooled internal standard labeled with Cy2. Spots were detected and normalized volume ratios were calculated using the Differential In-gel.

**Image acquisition and analysis**

After SDS-PAGE, the gels were scanned with a Typhoon 9400 fluorescence gel scanner (GE Healthcare, Piscataway, NJ) using appropriate individual excitation and emission wavelengths, filters and photomultiplier (PTM) values that are sensitive for each of the Cy3,Cy5 and Cy2 dyes (PTM values: 480nm, 490nm, 500nm, respectively).

Relative protein quantification was performed on LV samples (ICM and CNT) with DeCyder software v6.5 (GE Healthcare) and the multivariate statistical module EDA (Extended data analysis). The Differential in-gel analysis (DIA) module co-detected the 3 images of a gel (the internal standard and the two samples), measured the spot abundance in each image, and expressed these values as Cy3/Cy2 and Cy5/Cy2 ratios.

These DIA datasets were then analysed using the Biological Variation Analysis module (BVA), which enabled the spot maps to be matched and the Cy3/Cy2 and Cy5/Cy2 ratios to be compared. Only protein spots with >1.5-fold differences in abundance were considered for the analysis. A statistical analysis was then carried out to determine the changes in protein species, with P-values below 0.05 accepted as significant when the Student´s t-test was applied.

Finally, a multivariate analysis was performed by Principal Component Analysis (PCA) using the algorithm included in the EDA module of the DeCyder software (version 6.5) based on the spots that matched across all the gels. A pattern analysis hierarchical classification was obtained using the Pearson coefficient based on the spots present in 90% of all the gels.

**In-gel protein digestion**

Protein spots were excised manually and then automatically digested using the Ettan Digester (GE Healthcare). We used the digestion protocol previously described by Schevchenko *et al*. [1] with minor variations: the gel plugs were reduced using 10 mM DTT (Sigma-Aldrich; St. Louis, MO, USA) in 50 mM ammonium bicarbonate (99% purity; Scharlau) and alkylated using 55 mM iodoacetamide (Sigma-Aldrich) in 50 mM ammonium bicarbonate. The gel pieces were then rinsed with 50 mM ammonium bicarbonate in 50% methanol (gradient, high-performance liquid chromatography [HPLC] grade; Scharlau) and acetonitrile (gradient, HPLC grade; Scharlau) and dried in a Speedvac. Modified porcine trypsin (sequencing grade; Promega, Madison, WI, USA) at a final concentration of 20 ng/µL in 20 mM ammonium bicarbonate was added to the dry gel pieces, and the digestion was allowed to proceed at 37ºC overnight. Finally, 60% aqueous acetonitrile and 0.5% trifluoroacetic acid (99.5% purity; Sigma-Aldrich) were added to extract peptides.

**MALDI-MS(/MS) and database searching**

A total of 0.5 μL of each digestion solution was deposited using the thin-layer method onto a 384 Opti-TOF 123 × 81 mm MALDI plate (Applied Biosystems) and allowed to dry at RT. The same volume of matrix (3 mg/mL α-cyano-4-hydroxycinnamic acid (Sigma-Aldrich) in 60% acetonitrile/0.5% trifluoroacetic acid) was applied on every sample in the MALDI plate. MALDI-MS(/MS) data were obtained in an automated analysis loop by using a 4800 Plus MALDI TOF/TOF Analyzer (Applied Biosystems). Spectra were acquired in the reflector positive-ion mode by using an Nd:YAG 355-nm wavelength laser at 200 Hz laser frequency, and 1,000–2,000 individual spectra were averaged. The spectra were acquired uniformly by using fixed laser intensity. For the MS/MS 1-kV analysis mode, precursors were accelerated to 8 kV in Source 1 and selected using a relative resolution of 200 (FWHM) and metastable suppression. Fragment ions generated by collision with air in a CID chamber were further accelerated by 15 kV in Source 2. The mass data were automatically analyzed using the 4000 Series Explorer Software, Version 3.5.3 (Applied Biosystems). The MALDI-TOF mass spectra were internally calibrated using 2 trypsin autolysis ions with m/z = 842.510 and 2211.105. In the case of MALDI-MS/MS, calibrations were performed using the fragment-ion spectra obtained for Glub-fibrinopeptide (4700 Cal Mix; Applied Biosystems). MALDI-MS and MS/MS data were combined in the GPS Explorer Software Version 3.6 to search a non-redundant protein database (Swiss-Prot 2012_08) by using the Mascot software, version 2.2 (Matrix Science) [2], featuring 50 ppm precursor tolerance, 0.6-Da MS/MS fragment tolerance, carbamidomethyl cysteine as the fixed modification, and oxidized methionine as the variable modification, and allowing for 1 missed cleavage. The MALDI-MS (/MS) spectra and database search results were manually inspected in detail using the aforementioned software. In the case of the combined MS and MS/MS data, identifications were accepted when the confidence interval (CI%) calculated using the GPS software was ≥95%. Because Protein Scores and Ion Scores obtained from distinct searches cannot be directly compared, the GPS software calculates the CI% to combine the results of MS and MS/MS database searches. This coefficient value refers to a <5% probability of the observed match being a random event. In the case of the PMF spectra, identifications were accepted when the CI% was ≥99%.

**Selected reaction monitoring (SRM)**

Protein samples were reduced by incubating them with 100 mM DTT (Sigma Aldrich) in 50 mM ammonium bicarbonate (99% purity; Scharlau) for 30 min at 37ºC. After reduction, alkylation with 55 mM iodoacetamide (Sigma Aldrich) in 50 mM ammonium bicarbonate was conducted for 20 min at RT. Next, we added 50 mM ammonium bicarbonate, 15% acetonitrile (LCMS grade, Scharlau), and, finally, sequencing-grade modified porcine trypsin (Promega) at a final ratio of 1 µg trypsin: 50 µg protein. After digestion at 37 ºC overnight, 2% formic acid (99.5% purity; Sigma Aldrich) was added and samples were cleaned using Pep-Clean spin columns (Pierce) according to the manufacturer’s instructions. Tryptic digests were dried in a Speedvac and resuspended in 2% acetonitrile/2% formic acid prior to MS analysis.

The LC-MS/MS system consisted of a TEMPO nano LC system (Applied Biosystems) combined with a nano LC Autosampler and coupled to a modified triple quadrupole (Applied Biosystems 4000 QTRAP LC/MS/MS System). Three replicate injections (4 µL containing 8 µg of protein) were made for each sample (except 2 samples with only 1 injection per sample) by using mobile phase A (2% ACN/98% water, 0.1% FA) at a flow rate of 10 µL/min for 5 min. Peptides were loaded onto a µ-Precolumn Cartridge (Acclaim Pep Map 100 C18; 5 µm, 100Å; 300 µm i.d. × 5 mm, LC Packings) to preconcentrate and desalt samples. Reversed-phase LC was performed on a C18 column (Onyx Monolithic C18; 150 × 0.1 mm i.d., Phenomenex) in a gradient of phase A and phase B (98% ACN/2% water, 0.1% FA). Peptides were eluted at a flow rate of 900 nL/min by following these steps: 2–15% B for 2 min, 15–30% B for 18 min, 30–50% B for 5 min, 50–90% B for 2 min, and, finally, 90% B for 3 min. The column was then regenerated with 2% B for another 15 min. Both TEMPO nano LC and 4000 QTRAP system were controlled using the Analyst Software, v.1.4.5. The mass spectrometer was set to operate in the positive-ion mode with an ion-spray voltage of 2800 V and a nanoflow interface heater temperature of 150°C. Source gas 1 and curtain gas were set to 20 and 20 psi, respectively, and nitrogen was applied as both curtain and collision gases. Collision energy was optimized to obtain maximal transmission efficiency and sensitivity for each SRM transition. A total of 42 MRM transitions (3 per peptide) were monitored during the analysis of each sample and were acquired at unit resolution in both Q1 and Q3, with dwell times of 20 and 50 ms that resulted in a cycle time of 1.2303 s. The IntelliQuan algorithm included in the Analyst 1.4.5 software was used to calculate abundances based on the peak areas after integration.

**RNA extraction**

Heart samples were homogenized in TRIzol® reagent in a TissueLysser LT (Qiagen, UK). All RNA extractions were performed using a PureLink™ Kit according to the manufacturer’s instructions (Ambion Life Technologies, CA, USA). RNA was quantified using a NanoDrop1000 spectrophotometer (Thermo Fisher Scientific, UK), and the purity and integrity of the RNA samples were measured using an Agilent 2100 Bioanalyzer with an RNA 6000 Nano LabChip kit (Agilent Technologies, Spain). All samples showed a 260/280 ratio of >2.0 and an RNA integrity number of ≥9.

**RNAseq**

The RNA samples were isolated using a MicroPoly(A) Purist Kit™ (Ambion, USA). The total polyA-RNA samples were used to generate whole transcriptome libraries that were sequenced on a SOLiD 5500XL platform as per the manufacturer’s recommendations (Life Technologies, CA). The amplified cDNA quality was analyzed using the Bioanalyzer 2100 DNA 1000 kit (Agilent Technologies, Spain), and the cDNA was quantified using the Qubit 2.0 Fluorometer (Invitrogen, UK). Whole transcriptome libraries were used to generate SOLiD templated beads by following the SOLiD Templated Bead Preparation guide. Bead quality was estimated based on WFA (workflow analysis) parameters. The samples were sequenced using the 50625 paired-end protocol, which generated 75 nt + 35 nt (Paired-End) + 5 nt (Barcode) sequences. Quality data were measured using the SETS software parameters (SOLiD Experimental Tracking System).

**Computational analysis of RNAseq data**

The initial whole transcriptome paired-end reads obtained from the sequencing were mapped against the latest version of the human genome (Version GRchr37/hg19) by using the Life Technologies mapping algorithm (http://www.lifetechnologies.com/). The aligned records were reported in the BAM/SAM format [3]. Bad quality reads (Phred score <10) were eliminated using the Picard Tools software [4].

The isoform and gene predictions were subsequently estimated using the cufflinks method [5], and the expression levels were calculated using the HTSeq software [6]. The Edge method was applied to analyze the differential expression between conditions [7]. This method relies on a Poisson model to estimate the RNAseq data variance for differential expression. We selected genes and isoforms that were calculated to exhibit *P*<0.05 and fold-change >1.5.

**SUPPLEMENTAL REFERENCES**

1. **Schevchenko A, Wilm M, Vorm O, Mann M.** Mass spectrometric sequencing of proteins from silver stained polyacrylamide gels. *Anal Chem.* 1996; 68: 850-858.
2. **Perkins DN, Pappin DJ, Creasy DM, Cottrell JS**. Probability-based protein identification by searching sequence databases using mass spectrometry data. *Electrophoresis.* 1999; 20: 3551-3567.
3. **Li H, Handsaker B, Wysoker A, et al**. 1000 Genome Project Data Processing Subgroup. The Sequence Alignment/Map format and SAMtools. *Bioinformatics.* 2009; 25: 2078-2079.
4. **McKenna A, Hanna M, Banks E, et al.** The Genome Analysis Toolkit: a MapReduce framework for analyzing next-generation DNA sequencing data. *Genome Res.* 2010; 20: 1297-1303.
5. **Trapnell C, Williams BA, Pertea G, et al.** Transcript assembly and quantification by RNASeq reveals unannotated transcripts and isoform switching during cell differentiation. *Nat Biotechnol.* 2010; 28: 511-515.
6. **Anders S, Huber W.** Differential expression analysis for sequence count data. *Genome Biol.* 2010; 11: R106.
7. **Robinson MD, McCarthy DJ, Smyth GK.** edge R: A Bioconductor package for differential expression analysis of digital gene expression data. *Bioinformatics.* 2010; 26: 139-140.

**Supplemental Table 1:** Additional data on MS protein identification of ICM spots with differential expression by MALDI-MS.

| **S**  **Spot** | **N^a^ / %^b^** | **Mascot Score** | **Peptides identified by MS** | | **Identified protein** | **Accession code** | **Expected Mw** | **Expected pI** |
| --- | --- | --- | --- | --- | --- | --- | --- | --- |
|  |  |  | **M+H** | **Sequence** |  |  |  |  |
| 219 | 18/29 | 101 | 822.42 | K.ALTSFER.D | Trifunctional enzyme subunit alpha | ECHA_HUMAN | 83688 | 9.16 |
|  |  |  | 1012.46 | K.DGPGFYTTR.C |  |  |  |  |
|  |  |  | 1039.53 | R.FVDLYGAQK.I |  |  |  |  |
|  |  |  | 1202.59 | K.GFYIYQEGVK.R |  |  |  |  |
|  |  |  | 1317.68 | K.MQLLEIITTEK.T |  |  |  |  |
|  |  |  | 1333.70 | K.MQLLEIITTEK.T Oxidation (M) |  |  |  |  |
|  |  |  | 1337.60 | R.CLAPMMSEVIR.I 2 Oxidation (M) |  |  |  |  |
|  |  |  | 1397.72 | K.LTAYAMTIPFVR.Q Oxidation (M |  |  |  |  |
|  |  |  | 1407.67 | K.VIGMHYFSPVDK.M Oxidation (M) |  |  |  |  |
|  |  |  | 1410.62 | K.SEVSSDEDIQFR.L |  |  |  |  |
|  |  |  | 1576.77 | K.MVGVPAALDMMLTGR.S Oxidation (M) |  |  |  |  |
|  |  |  | 1625.83 | R.TIEYLEEVAITFAK.G |  |  |  |  |
|  |  |  | 1629.82 | K.TLQEVTQLSQEAQR.I |  |  |  |  |
|  |  |  | 1694.83 | K.ADMVIEAVFEDLSLK.H Oxidation (M) |  |  |  |  |
|  |  |  | 1841.79 | K.TGIEQGSDAGYLCESQK.F |  |  |  |  |
|  |  |  | 2006.10 | K.TVLGTPEVLLGALPGAGGTQR.L |  |  |  |  |
|  |  |  | 2060.96 | R.DSIFSNLTGQLDYQGFEK.A |  |  |  |  |
|  |  |  | 2289.20 | K.MGLVDQLVEPLGPGLKPPEER.T Oxidation (M) |  |  |  |  |
| 344 | 29/52 | 270 | 806.42 | R.FGILTEK.Y | 60 kDa heat shock protein, mitochondrial | ETFD_HUMAN | 69250 | 7.31 |
|  |  |  | 818.42 | K.NSWVWK.E |  |  |  |  |
|  |  |  | 933.46 | K.ELFPDWK.E |  |  |  |  |
|  |  |  | 960.48 | R.GMEPWTLK.H |  |  |  |  |
|  |  |  | 1019.45 | R.WEGVNMER.F |  |  |  |  |
|  |  |  | 1030.53 | K.ELWVIDEK.N |  |  |  |  |
|  |  |  | 1035.45 | R.WEGVNMER.F + Oxidation (M) |  |  |  |  |
|  |  |  | 1114.60 | K.GIATNDVGIQK.D |  |  |  |  |
|  |  |  | 1190.60 | K.ELFPDWKEK.G |  |  |  |  |
|  |  |  | 1259.65 | R.ALNEGGFQSIPK.L |  |  |  |  |
|  |  |  | 1263.66 | R.ITTHYTIYPR.D |  |  |  |  |
|  |  |  | 1268.63 | K.GAPLNTPVTEDR.F |  |  |  |  |
|  |  |  | 1278.70 | K.QLAVAHEKDIR.V |  |  |  |  |
|  |  |  | 1290.62 | R.NLSIYDGPEQR.F |  |  |  |  |
|  |  |  | 1293.64 | R.VDHTVGWPLDR.H |  |  |  |  |
|  |  |  | 1427.77 | K.HHPSIRPTLEGGK.R |  |  |  |  |
|  |  |  | 1449.70 | R.ANCEPQTYGIGLK.E |  |  |  |  |
|  |  |  | 1483.70 | R.LQINAQNCVHCK.T |  |  |  |  |
|  |  |  | 1538.77 | K.VTIFAEGCHGHLAK.Q |  |  |  |  |
|  |  |  | 1630.82 | K.TIGLHVTEYEDNLK.N |  |  |  |  |
|  |  |  | 1741.90 | R.WKHHPSIRPTLEGGK.R |  |  |  |  |
|  |  |  | 1983.98 | K.AAQIGAHTLSGACLDPGAFK.E |  |  |  |  |
|  |  |  | 2057.08 | K.GAPLNTPVTEDRFGILTEK.Y |  |  |  |  |
|  |  |  | 2098.10 | R.FAEEADVVIVGAGPAGLSAAVR.L |  |  |  |  |
|  |  |  | 2120.07 | K.LTFPGGLLIGCSPGFMNVPK.I + Oxidation (M) |  |  |  |  |
|  |  |  | 2172.99 | R.FCPAGVYEFVPVEQGDGFR.L |  |  |  |  |
|  |  |  | 2229.19 | R.IPVPILPGLPMNNHGNYIVR.L + Oxidation (M) |  |  |  |  |
|  |  |  | 2249.14 | K.SGILAAESIFNQLTSENLQSK.T |  |  |  |  |
|  |  |  | 2287.08 | R.GLELHAKVTIFAEGCHGHLAK.Q |  |  |  |  |
| 348 | 5/13 | 93 | 908.46 | K.FPFAANSR.A | Dihydrolipoyl dehydrogenase, mitochondrial | DLDH_HUMAN | 54713 | 7.95 |
|  |  |  | 1523.73 | R.VCHAHPTLSEAFR.E |  |  |  |  |
|  |  |  | 1566.82 | K.NLGLEELGIELDPR.G |  |  |  |  |
|  |  |  | 1716.89 | K.AEVITCDVLLVCIGR.R |  |  |  |  |
|  |  |  | 1985.02 | K.MVVIGAGVIGVELGSVWQR.L + Oxidation (M) |  |  |  |  |
| 361 | 12/14 | 79 | 803.42 | R.FHTFPR.L | Delta-1-pyrroline-5-carboxylate dehydrogenase, mitochondrial | AL4A1_HUMAN | 62137 | 8.28 |
|  |  |  | 882.48 | R.LLEEHSR.I |  |  |  |  |
|  |  |  | 955.48 | K.NFHFVHR.S |  |  |  |  |
|  |  |  | 985.46 | R.SAFEYGGQK.C |  |  |  |  |
|  |  |  | 1225.56 | R.NAAGNFYINDK.S |  |  |  |  |
|  |  |  | 1318.67 | R.SADVESVVSGTLR.S |  |  |  |  |
|  |  |  | 1460.74 | K.STGSIVGQQPFGGAR.A |  |  |  |  |
|  |  |  | 1681.85 | R.ASGTNDKPGGPHYILR.W |  |  |  |  |
|  |  |  | 1713.87 | K.VANEPVLAFTQGSPER.D |  |  |  |  |
|  |  |  | 1727.86 | K.QVAQNLDRFHTFPR.L |  |  |  |  |
|  |  |  | 2269.16 | K.VANEPVLAFTQGSPERDALQK.A |  |  |  |  |
|  |  |  | 2534.28 | K.YAVELEGQQPISVPPSTNSTVYR.G |  |  |  |  |
| 407 | 17/47 | 114 | 908.45 | K.FPFAANSR.A | Dihydrolipoyl dehydrogenase, mitochondrial | DLDH_HUMAN | 54713 | 7.95 |
|  |  |  | 911.53 | R.GRIPVNTR.F |  |  |  |  |
|  |  |  | 1126.65 | K.ALTGGIAHLFK.Q |  |  |  |  |
|  |  |  | 1523.73 | R.VCHAHPTLSEAFR.E |  |  |  |  |
|  |  |  | 1566.83 | K.NLGLEELGIELDPR.G |  |  |  |  |
|  |  |  | 1580.76 | K.SEEQLKEEGIEYK.V |  |  |  |  |
|  |  |  | 1716.89 | K.AEVITCDVLLVCIGR.R |  |  |  |  |
|  |  |  | 1770.82 | K.ALLNNSHYYHMAHGK.D + Oxidation (M) |  |  |  |  |
|  |  |  | 1818.86 | K.NETLGGTCLNVGCIPSK.A |  |  |  |  |
|  |  |  | 1969.08 | K.MVVIGAGVIGVELGSVWQR.L |  |  |  |  |
|  |  |  | 1978.07 | K.IPNIYAIGDVVAGPMLAHK.A |  |  |  |  |
|  |  |  | 1985.08 | K.MVVIGAGVIGVELGSVWQR.L + Oxidation (M) |  |  |  |  |
|  |  |  | 1994.06 | K.IPNIYAIGDVVAGPMLAHK.A + Oxidation (M) |  |  |  |  |
|  |  |  | 2513.30 | R.LGADVTAVEFLGHVGGVGIDMEISK.N |  |  |  |  |
|  |  |  | 2529.30 | R.LGADVTAVEFLGHVGGVGIDMEISK.N + Oxidation (M) |  |  |  |  |
|  |  |  | 3358.79 | K.NILIATGSEVTPFPGITIDEDTIVSSTGALSLK.K |  |  |  |  |
|  |  |  | 3369.68 | R.VLGAHILGPGAGEMVNEAALALEYGASCEDIAR.V + Oxidation (M) |  |  |  |  |
| 464 |  | 165 | 844.49 | R.STVAQLVK.R | ATP synthase subunit alpha , mitochondrial | ATPA_HUMAN | 59828 | 9.16 |
|  |  |  | 891.49 | K.LELAQYR.E |  |  |  |  |
|  |  |  | 999.53 | R.VLSIGDGIAR.V |  |  |  |  |
|  |  |  | 1105.55 | R.NALGSSFIAAR.N |  |  |  |  |
|  |  |  | 1286.68 | K.HALIIYDDLSK.Q |  |  |  |  |
|  |  |  | 1552.73 | R.EAYPGDVFYLHSR.L |  |  |  |  |
|  |  |  | 1574.77 | R.ILGADTSVDLEETGR.V |  |  |  |  |
|  |  |  | 1623.88 | R.TGAIVDVPVGEELLGR.V |  |  |  |  |
|  |  |  | 2229.18 | R.VHGLRNVQAEEMVEFSSGLK.G |  |  |  |  |
|  |  |  | 2337.14 | R.EVAAFAQFGSDLDAATQQLLSR.G |  |  |  |  |
|  |  |  | 2366.25 | K.FENAFLSHVVSQHQALLGTIR.A |  |  |  |  |
| 472 | 9/20 | 164 | 834.46 | R.EAVTFLR.K | Dihydrolipoyllysine-residue succinyltransferase component of 2-oxoglutarate dehydrogenase complex, mitochondrial | ODO2_HUMAN | 49067 | 9.11 |
|  |  |  | 851.56 | R.GLVVPVIR.N |  |  |  |  |
|  |  |  | 889.48 | R.TITELGEK.A |  |  |  |  |
|  |  |  | 1188.65 | K.VEGGTPLFTLR.K |  |  |  |  |
|  |  |  | 1407.65 | R.NVEAMNFADIER.T |  |  |  |  |
|  |  |  | 1418.73 | R.DYIDISVAVATPR.G |  |  |  |  |
|  |  |  | 1423.64 | R.NVEAMNFADIER.T + Oxidation (M) |  |  |  |  |
|  |  |  | 1477.71 | K.TPAFAESVTEGDVR.W |  |  |  |  |
|  |  |  | 2216.13 | K.ASAFALQEQPVVNAVIDDTTK.E |  |  |  |  |
| 614 | 11/28 | 74 | 1070.55 | R.GLSLPPACTR.A | Creatine kinase S-type, mitochondrial | KCRS_HUMAN | 47988 | 8.46 |
|  |  |  | 1078.61 | K.VPPPLPQFGK.K |  |  |  |  |
|  |  |  | 1215.61 | R.HNGYDPRVMK.H |  |  |  |  |
|  |  |  | 1322.73 | MASIFSKLLTGR.N |  |  |  |  |
|  |  |  | 1389.70 | R.LFPPSADYPDLR.K |  |  |  |  |
|  |  |  | 1517.79 | R.LFPPSADYPDLRK.H |  |  |  |  |
|  |  |  | 1672.82 | K.TFLIWINEEDHTR.V |  |  |  |  |
|  |  |  | 1733.90 | R.LGYILTCPSNLGTGLR.A |  |  |  |  |
|  |  |  | 1778.86 | K.ITQGQFDEHYVLSSR.V |  |  |  |  |
|  |  |  | 2108.00 | R.GTGGVDTAAVADVYDISNIDR.I |  |  |  |  |
|  |  |  | 2264.05 | K.RGTGGVDTAAVADVYDISNIDR.I |  |  |  |  |
| 689 | 3/9 | 52 | 814.49 | R.GLAVEAKK.T | Elongation factor Tu, mitochondrial | EFTU_HUMAN | 49852 | 7.26 |
|  |  |  | 1541.85 | K.LLDAVDTYIPVPAR.D |  |  |  |  |
|  |  |  | 2128.16 | R.DLEKPFLLPVEAVYSVPGR.G |  |  |  |  |
| 882 | 8/20 | 74 | 844.51 | R.STVAQLVK.R | ATP synthase subunit alpha, mitochondrial | ATPA_HUMAN | 59828 | 9.16 |
|  |  |  | 891.51 | K.LELAQYR.E |  |  |  |  |
|  |  |  | 1105.55 | R.NALGSSFIAAR.N |  |  |  |  |
|  |  |  | 1286.67 | K.HALIIYDDLSK.Q |  |  |  |  |
|  |  |  | 1552.73 | R.EAYPGDVFYLHSR.L |  |  |  |  |
|  |  |  | 2229.18 | R.VHGLRNVQAEEMVEFSSGLK.G |  |  |  |  |
|  |  |  | 2308.14 | K.QGQYSPMAIEEQVAVIYAGVR |  |  |  |  |
|  |  |  | 2337.14 | R.EVAAFAQFGSDLDAATQQLLSR.G |  |  |  |  |
| 960 | 12/33 | 125 | 816.41 | K.ELDRER.A | Coiled-coil-helix-coiled-coil-helix domain-containing protein 3, mitochondrial | CHCH3_HUMAN | 26421 | 8.48 |
|  |  |  | 851.43 | K.ILQCYR.E |  |  |  |  |
|  |  |  | 972.50 | R.AAANEQLTR.A |  |  |  |  |
|  |  |  | 1199.64 | R.VAEELALEQAK.K |  |  |  |  |
|  |  |  | 1516.70 | R.YESHPVCADLQAK.I |  |  |  |  |
|  |  |  | 1574.71 | R.YSGAYGASVSDEELK.R |  |  |  |  |
|  |  |  | 1706.84 | R.VTFEADENENITVVK.G |  |  |  |  |
|  |  |  | 1862.94 | R.RVTFEADENENITVVK.G |  |  |  |  |
| 1019 | 7/42 | 145 | 832.44 | R.SVEETLR.L | Thioredoxin-dependent peroxide reductase, mitochondrial oxoglutarate dehydrogenase complex, mitochondrial | PRDX3_HUMAN | 28017 | 7.67 |
|  |  |  | 1205.65 | K.HLSVNDLPVGR.S |  |  |  |  |
|  |  |  | 1284.74 | R.GLFIIDPNGVIK.H |  |  |  |  |
|  |  |  | 1461.78 | R.DYGVLLEGSGLALR.G |  |  |  |  |
|  |  |  | 1565.73 | R.TSLTNLLCSGSSQAK.L |  |  |  |  |
|  |  |  | 1953.94 | K.GTAVVNGEFKDLSLDDFK.G |  |  |  |  |
|  |  |  | 3384.64 | K.AFQYVETHGEVCPANWTPDSPTIKPSPAASK.E |  |  |  |  |
|  | 6/33 | 80 | 1237.68 | K.VAEVLQVPPMR.V | NADH dehydrogenase [ubiquinone] flavoprotein 2, mitochondrial | NDUV2_HUMAN | 27659 | 8.22 |
|  |  |  | 1253.67 | K.VAEVLQVPPMR.V + Oxidation (M) |  |  |  |  |
|  |  |  | 1335.79 | K.AAAVLPVLDLAQR.Q |  |  |  |  |
|  |  |  | 1671.76 | R.VYEVATFYTMYNR.K + Oxidation (M) |  |  |  |  |
|  |  |  | 2396.05 | R.DTPENNPDTPFDFTPENYKR.I |  |  |  |  |
|  |  |  | 2673.31 | R.FSCEPAGGLTSLTEPPKGPGFGVQAGL |  |  |  |  |
|  |  |  |  |  |  |  |  |  |
|  | | | |  |  |  |  |  |
|  | | | | |  |  |  |  |

**Supplemental Table 2:** Additional data on selected reaction monitoring (SRM) analysis.

| Accession code | Accession nº | Peptide Sequence | Q1 | Q3 | CE | Dwell time | Charge state | Fragment Ion |
| --- | --- | --- | --- | --- | --- | --- | --- | --- |
| ATPA_HUMAN | P25705 | AVDSLVPIGR | 513.80 | 541.35 | 27.61 | 20 | +2 | y5 |
| ATPA_HUMAN | P25705 | AVDSLVPIGR | 513.80 | 654.43 | 28.00 | 20 | +2 | y6 |
| ATPA_HUMAN | P25705 | AVDSLVPIGR | 513.80 | 856.49 | 28.00 | 20 | +2 | y8 |
| ATPA_HUMAN | P25705 | TGAIVDVPVGEELLGR | 812.95 | 969.54 | 41.00 | 20 | +2 | y9 |
| ATPA_HUMAN | P25705 | TGAIVDVPVGEELLGR | 812.95 | 1068.60 | 41.00 | 20 | +2 | y10 |
| ATPA_HUMAN | P25705 | TGAIVDVPVGEELLGR | 812.95 | 1183.63 | 40.77 | 20 | +2 | y11 |
| DLDH_HUMAN | P09622 | ISHGLQGLSAVPLR | 724.42 | 642.39 | 36.87 | 20 | +2 | y6 |
| DLDH_HUMAN | P09622 | ISHGLQGLSAVPLR | 724.42 | 812.50 | 37.00 | 20 | +2 | y8 |
| DLDH_HUMAN | P09622 | ISHGLQGLSAVPLR | 724.42 | 940.56 | 37.00 | 20 | +2 | y9 |
| DLDH_HUMAN | P09622 | NLGLEELGIELDPR | 784.42 | 799.43 | 39.51 | 20 | +2 | y7 |
| DLDH_HUMAN | P09622 | NLGLEELGIELDPR | 784.42 | 1041.56 | 40.00 | 20 | +2 | y9 |
| DLDH_HUMAN | P09622 | NLGLEELGIELDPR | 784.42 | 1170.60 | 39.51 | 20 | +2 | y10 |
| EFTU_HUMAN | P49411 | AEAGDNLGALVR | 593.31 | 515.33 | 31.11 | 20 | +2 | y5 |
| EFTU_HUMAN | P49411 | AEAGDNLGALVR | 593.31 | 742.46 | 31.00 | 20 | +2 | y7 |
| EFTU_HUMAN | P49411 | AEAGDNLGALVR | 593.31 | 985.54 | 31.00 | 20 | +2 | y10 |
| EFTU_HUMAN | P49411 | LLDAVDTYIPVPAR | 771.93 | 539.33 | 38.96 | 20 | +2 | y5 |
| EFTU_HUMAN | P49411 | LLDAVDTYIPVPAR | 771.93 | 652.41 | 38.96 | 20 | +2 | y6 |
| EFTU_HUMAN | P49411 | LLDAVDTYIPVPAR | 771.93 | 1031.55 | 39.00 | 20 | +2 | y9 |
| PRDX3_HUMAN | P30048 | GLFIIDPNGVIK | 643.38 | 627.38 | 33.31 | 20 | +2 | y6 |
| PRDX3_HUMAN | P30048 | GLFIIDPNGVIK | 643.38 | 742.41 | 33.31 | 20 | +2 | y7 |
| PRDX3_HUMAN | P30048 | GLFIIDPNGVIK | 643.38 | 855.49 | 33.00 | 20 | +2 | y8 |
| PRDX3_HUMAN | P30048 | DYGVLLEGSGLALR | 731.90 | 673.40 | 37.20 | 20 | +2 | y7 |
| PRDX3_HUMAN | P30048 | DYGVLLEGSGLALR | 731.90 | 915.53 | 37.00 | 20 | +2 | y9 |
| PRDX3_HUMAN | P30048 | DYGVLLEGSGLALR | 731.90 | 1028.61 | 37.00 | 20 | +2 | y10 |
